# Structure-Based Mutations in the Herpes Simplex Virus 1 Glycoprotein B Ectodomain Arm Impart a Slow-Entry Phenotype

**DOI:** 10.1128/mBio.00614-17

**Published:** 2017-05-16

**Authors:** Qing Fan, Sarah J. Kopp, Sarah A. Connolly, Richard Longnecker

**Affiliations:** a Department of Microbiology-Immunology, Feinberg School of Medicine of Northwestern University, Chicago, Illinois, USA; b Department of Health Sciences, Department of Biological Sciences, DePaul University, Chicago, Illinois, USA; Icahn School of Medicine at Mount Sinai

## Abstract

Glycoprotein B (gB) is the conserved herpesvirus fusion protein, and it is required for the entry of herpesviruses. The structure of the postfusion conformation of gB has been solved for several herpesviruses; however, the gB prefusion crystal structure and the details of how the protein refolds from a prefusion to a postfusion form to mediate fusion have not been determined. Using structure-based mutagenesis, we previously reported that three mutations (I671A, H681A, and F683A) in the C-terminal arm of the gB ectodomain greatly reduced cell-cell fusion. This fusion deficit could be rescued by the addition of a hyperfusogenic mutation, suggesting that the gB triple mutant was not misfolded. Using a bacterial artificial chromosome (BAC), we constructed two independent herpes simplex virus 1 mutant strains (gB 3A) carrying the three arm mutations. The gB 3A viruses have 200-fold smaller plaques than the wild-type virus and demonstrate remarkably delayed entry into cells. Single-step and multistep growth curves show that gB 3A viruses have delayed replication kinetics. Interestingly, incubation at 40°C promoted the entry of the gB 3A viruses. We propose that the gB 3A viruses’ entry deficit is due to a loss of interactions between residues in the gB C-terminal arm and the coiled-coil core of gB. The results suggest that the triple alanine mutation may destabilize the postfusion gB conformation and/or stabilize the prefusion gB conformation and that exposure to elevated temperatures can overcome the defect in gB 3A viruses.

## IMPORTANCE

Because of its complexity, the mechanism of herpesvirus entry into cells is not well understood. Our study investigated one of the most important unanswered questions about herpesvirus entry; i.e., how does the herpesvirus fusion protein gB mediate membrane fusion? gB is an essential protein that is conserved in all herpesviruses and is thought to undergo a conformational change to provide the energy to fuse the viral and cellular membranes. Using our understanding of the structure of gB, we designed mutations in the gB “arm” region that we predicted would impede gB function. We introduced these mutations into herpes simplex virus 1 by using a bacterial artificial chromosome, and the mutant virus exhibited a drastically delayed rate of entry. This entry defect was rescued by incubation at elevated temperatures, supporting a hypothesis that the engineered mutations altered the energetics of gB refolding. This study supports our hypothesis that an interaction between the gB arm and the core of gB is critical for gB refolding and the execution of membrane fusion, thus providing key details about the function of gB in herpesvirus-mediated fusion and subsequent virus entry.

## INTRODUCTION

Herpes simplex virus 1 (HSV-1) entry into cells and virus-induced cell-cell fusion require the coordinated action of viral entry glycoprotein D (gD), gH, gL, and gB. The binding of gD to one of several host receptors results in a conformational change in gD that is proposed to signal the gH/gL heterodimer to trigger the fusogenic activity of gB (1–3). Entry receptors that bind to gD include herpesvirus entry mediator (HVEM) (4), nectin-1 (5, 6), nectin-2 (7, 8), and modified heparan sulfate (HS) (9, 10). Another receptor, paired immunoglobulin-like type 2 receptor (PILRα), binds to gB and can mediate viral entry, provided that gD also binds to a receptor (11).

Fusion of the viral membrane with the host cell membrane is executed by gB, a class III viral fusion protein (12) that is conserved across all herpesviruses (13). Viral fusion proteins initially fold to a metastable prefusion state. Upon triggering, the proteins insert themselves into the cellular membrane and refold from a prefusion to a postfusion conformation to bring the viral and cell membranes together. Crystal structures of the postfusion gB conformation have been solved for three herpesviruses (12, 14–16). The prefusion conformation of gB and the details of how it refolds to execute fusion are unclear. Attempts to capture a stable prefusion form of HSV-1 gB for crystallization have been unsuccessful (17).

**FIG 1:**
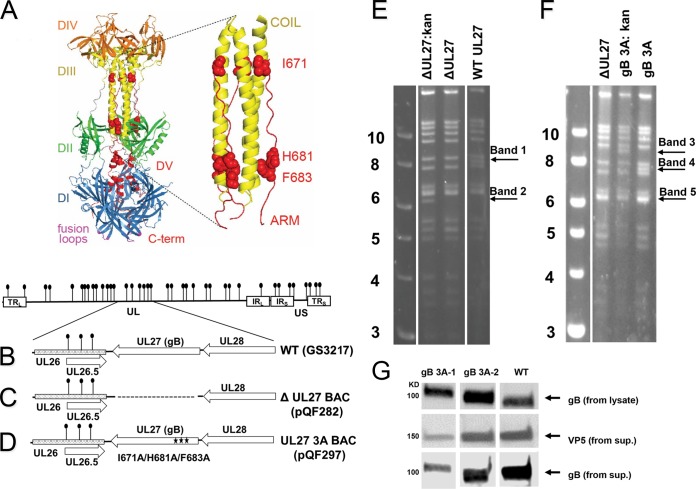
Generation of gB 3A viruses. (A) HSV-1 gB ectodomain (PDB ID 2GUM). The five domains (DI to DV) of HSV-1 gB are color coded. The fusion loops (purple) are located on the same side of the molecule as the C-terminal region (red), giving a postfusion hairpin conformation. The domain V arm (red) packs against the coiled-coil core of domain III (yellow) in an antiparallel orientation. A closeup of the coil-arm region is shown, with the three residues that are mutated in gB 3A shown as red spheres. (B) HSV-1 genome with internal repeats (IR) and terminal repeats (TR) shown as white rectangles. BamHI sites are indicated by lollipops. A region of the genome is expanded to show the UL27 open reading frame (gB opening reading frame) and the neighboring UL26 and UL28 open reading frames (arrowheads indicate gene orientation). (C) HSV-1 genome with UL27 (gB) deletion. Dotted lines indicate the UL27 open reading frame deletion in BAC pQF282. (D) gB 3A viruses were made by recombining the gB open reading frame from plasmid pSG5-HSVgB-I671A/H681A/F683A into the gB-null BAC to create BAC pQF297. (E, F) Ethidium bromide-stained agarose gels of BamHI-digested BAC DNA. Digestion of the parental, intermediate, and final BACs generated to create gB-null BAC pQF282 (E) and gB 3A BAC pQF297 (F) are shown. The Kan^r^ cassette is ~1.0 kb and contains one BamHI site. Removal of the Kan^r^ cassette changes the BamHI digestion pattern, as highlighted by the arrows. Samples shown together were run on a single gel, but the lanes were rearranged for clarity. (G) Incorporation of gB into virions. Cells were infected with WT (G3217) or gB 3A viruses and cell lysates or supernatants (sup.) were harvested. Supernatants were pelleted through 10% sucrose. As shown at the top, lysates were separated by SDS-PAGE, blotted, and probed for HSV-1 gB. As shown at the bottom, duplicate supernatant samples were separated by SDS-PAGE, blotted, and probed for VP5 or gB. Samples shown next to one another were run on a single gel, but the lanes were rearranged for clarity. The values to the left of panels E and F identify the kilobase pairs of the DNA ladder.

gB is trimeric and consists of five domains (Fig. 1A). The postfusion gB structure adopts a hairpin conformation wherein the hydrophobic fusion loops that insert themselves into the host cell membrane reside at the same end of the molecule as the C terminus of the ectodomain, the site where the transmembrane domain would connect. This hairpin arrangement is common for the postfusion form of fusion proteins. A central coiled-coil core consisting of three α-helices in domain III contributes to the stability of the trimer. The C-terminal region of the ectodomain (domain V) consists of a long arm that extends down the length of the molecule and packs into a groove in the central coiled coil. The antiparallel packing of this arm against the coiled-coil helices is reminiscent of the six-helix bundle present in the postfusion conformation of class I fusion proteins. Formation of the class I six-helix bundle is proposed to help overcome the energy barrier to membrane fusion (18). We hypothesize that gB refolds similarly to class I fusion proteins and that the arm packing against the coiled coil provides a driving force for the gB conformational change from its prefusion to its postfusion form. Interestingly, electron cryotomography of gB on vesicles revealed a gB conformation that lacks the coil-arm complex, supporting the concept that this complex may be absent from the prefusion form (19).

Our previous mutagenesis study demonstrated that fusion was impaired by mutations in the domain V arm that were predicted to disrupt the coil-arm interaction (20). This phenotype could be rescued by the addition of a hyperfusogenic mutation in the gB cytoplasmic tail, suggesting that the reduced fusion phenotype was not due to global misfolding. When three alanine substitutions were made simultaneously in the gB arm (I671A, H681A, and F683A; gB 3A here), fusion was greatly reduced in a cell-cell fusion assay. If the interaction of the arm with the coil contributes to the conversion from a prefusion to a postfusion form, the gB 3A mutations may energetically favor the prefusion state of gB.

To further investigate the role of the gB arm region in virus entry, we generated and characterized HSV-1 strains carrying the gB 3A mutations. Addition of the gB 3A mutations to the viral genome permitted analysis of multistep growth curves and plaque sizes, data that cannot be collected by using a virus that is complemented phenotypically. Our results showed that a gB 3A mutant virus had impaired growth and significantly delayed penetration of cells. We found that elevated temperatures could partially rescue the penetration of cells by gB 3A virus. The ability of heat to overcome the penetration defect is consistent with the hypothesis that the gB 3A mutations favor the prefusion conformation. The mutations did not impact virus binding to cells, and increasing the expression of gH/gL did not compensate for the gB 3A fusion defect in a cell-cell fusion assay.

## RESULTS

### Construction of HSV-1 containing the gB 3A mutations.

To create an HSV-1 bacterial artificial chromosome (BAC) carrying the gB 3A mutations, UL27 (the gene encoding gB) was deleted from the HSV-1 BAC pGS3217 to generate gB-null BAC pQF282 (Fig. 1B and C). The gB 3A-encoding gene was then recombined into BAC pQF282 to generate BAC pQF297 (Fig. 1D). At each step of these constructions, intermediate BACs (with kanamycin resistance [Kan^r^]-encoding gene insertions) and final BACs (with the Kan^r^-encoding gene insertions removed) were confirmed by at least four restriction enzyme digestions. For example, BamHI digestions of the BACs show the expected band shifts for pQF282 and pQF297 (Fig. 1E and F). Band 1 is present only in the wild-type (WT) BAC, and band 2 is present only in the intermediate BAC with Kan^r^-encoding gene insertions but not in the BAC with UL27 deleted or the WT BAC (Fig. 1E). Similarly, band 3 is present only in the intermediate BAC with Kan^r^-encoding gene insertions, band 4 is present only in the final gB 3A BAC, and band 5 (consisting of two bands that migrate as one because of the similarity of their molecular weights) is present only in the WT BAC and the BAC with UL27 deleted (Fig. 1F).

The pGS3217 and pQF297 BACs were transfected into Vero-Cre cells, and virus was harvested from the cells. Vero cells were infected with samples from these transfections to generate GS3217 (WT) and gB 3A virus stocks. Two distinct gB 3A virus stocks (gB 3A-1 and gB 3A-2) were created by independent transfections of BAC pQF297 into Vero-Cre cells. To confirm the expression of the mutant gB proteins and gB incorporation into the virions, Western blot assays were performed (Fig. 1G). Vero cells were infected with the gB 3A viruses and the GS3217 (WT) virus at 0.01 PFU/cell. Because of the growth defects of gB 3A viruses (described below), infected cells and supernatants were harvested 7 days postinfection with the gB 3A viruses and 3 days postinfection with the G3217 (WT) virus. A Western blot assay of infected cell lysates probed with anti-gB serum R74 shows a band at the expected size for WT gB for all three viruses (Fig. 1G, top). To confirm that gB was incorporated into the gB 3A virions, virus was harvested from culture supernatants by centrifugation through a 10% sucrose cushion. Western blot assays of virus released into the medium demonstrated that mutant gB was incorporated into virions (Fig. 1G, middle and bottom).

### gB 3A viruses exhibit a growth delay.

**FIG 2:**
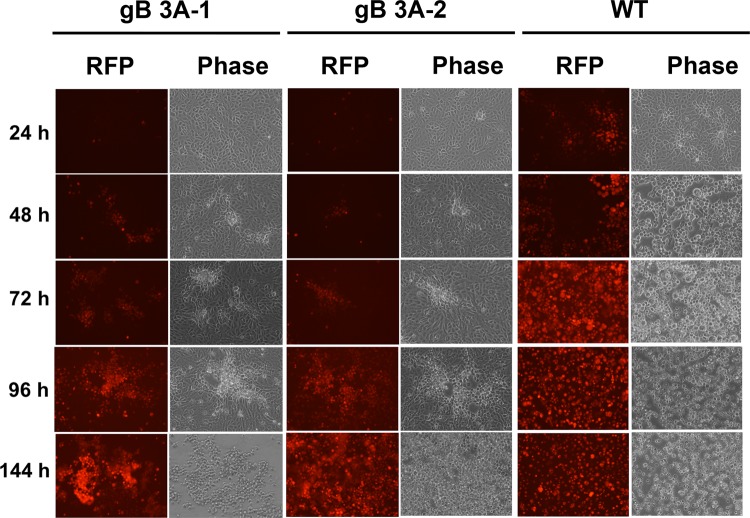
HSV-1 gB 3A cell infection. Vero cells plated in six-well plates were infected with gB 3A-1, gB 3A-2, or WT (G3217) HSV-1 at an MOI of 0.01. After 24, 48, 72, 96, or 144 h, cells were imaged for the RFP encoded by the virus with an EVOS Cell Imaging System at identical settings under ×20 magnification.

GS3217 (WT), gB 3A-1, and gB 3A-2 viruses encode tdTomato, allowing infection to be monitored by fluorescence microscopy. Vero cells were infected with viruses at 0.01 PFU/cell and incubated for 24 to 144 h before imaging. By microscopic examination at 24 h postinfection (hpi), 30 to 50% of the cells infected with GS3217 (WT) virus were red, whereas very few red cells were observed with both the gB 3A-1 and gB 3A-2 viruses (Fig. 2). At 48 hpi, all of the cells infected with GS3217 (WT) virus were red and the cytopathic effect (CPE) of infection was widespread. The cells infected with the gB 3A viruses did not achieve this level of infection and CPE until 4 to 6 days postinfection (Fig. 2).

### gB 3A viruses display a small-plaque phenotype.

**FIG 3:**
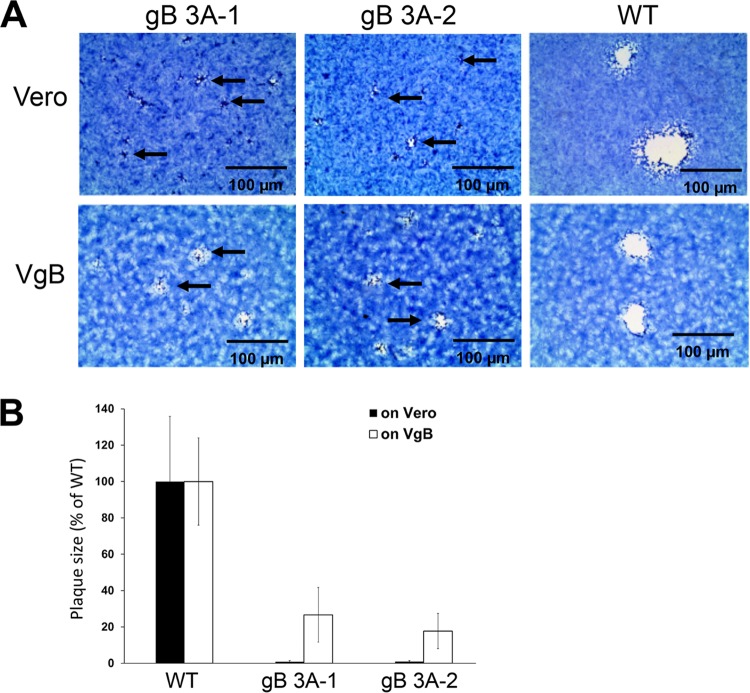
Plaque morphology of gB 3A viruses. Vero or VgB cells (Vero cells that express WT gB) were infected with gB 3A-1, gB 3A-2, or WT (G3217) HSV-1 at an MOI of 0.01. (A) Cells were stained with Giemsa stain 3 days after infection, and plaques were photographed with an EVOS Cell Imaging System at identical settings under ×4 magnification (scale bars, 100 µm). The arrows indicate small plaques. (B) gB 3A and WT plaque sizes on Vero or VgB cells were calculated by measuring the radii of at least 20 plaques from each virus, and the area of each plaque was determined. The gB 3A plaque sizes are presented as a percentage of the WT plaque area on Vero or VgB (gB-expressing) cells.

Compared to the GS3217 (WT) virus, both gB 3A viruses formed smaller plaques on Vero cells after 3 days of incubation (Fig. 3A). Relative to the GS3217 (WT) gB plaques, the gB 3A plaques were approximately 200-fold smaller, with a 50- to 300-fold range of reduction (Fig. 3B). The plaque size was partially restored when VgB cells (Vero-24 cells expressing WT gB) were infected with the gB 3A viruses (Fig. 3A), with gB 3A plaques sizes only two to three times smaller than those of the WT (Fig. 3B). This partial restoration of plaque size suggests that the mutations in the gB-encoding gene of gB 3A are responsible for the small-plaque phenotype. Notably, GS3217 (WT) virus formed larger plaques than gB 3A virus on both Vero and VgB cells. Although VgB cells express WT gB during gB 3A infection, the GS3217 (WT) virus may provide a greater level of WT gB during infection and this virus-encoded WT gB may contribute to the larger WT plaque size seen in VgB cells.

### gB 3A viruses have slow growth kinetics.

**FIG 4:**
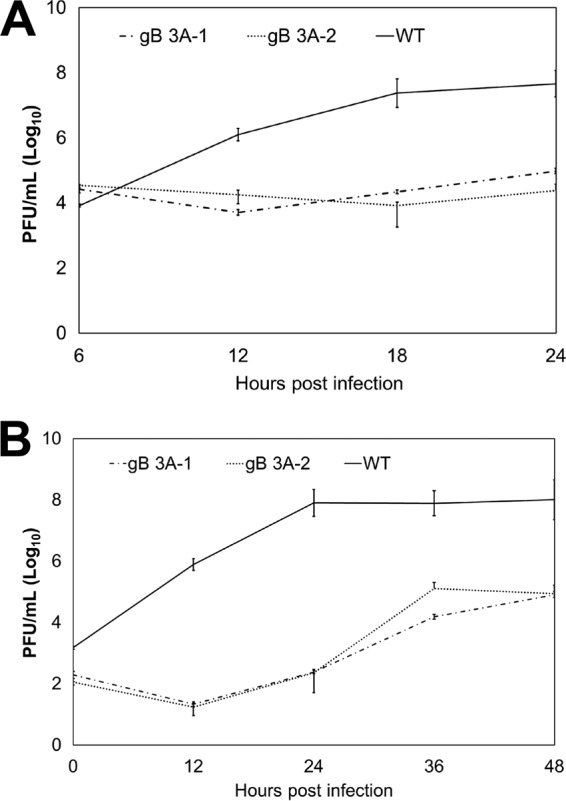
Analyses of gB 3A virus growth curves. (A) Single-step growth curve. Vero cells in six-well plates were infected with WT (GS3217), gB 3A-1, or gB 3A-2 virus at 5 PFU/cell. At the time points indicated, virus was harvested from total cell lysates. Viral titers were determined by a plaque assay on Vero cells. (B) Multistep growth curve. Vero cells in six-well plates were infected with WT (GS3217), gB 3A-1, or gB 3A-2 virus at 0.01 PFU/cell. At the time points indicated, virus was harvested from total cell lysates. Viral titers were determined by a plaque assay on Vero cells. Both graphs represent the average results of three independent experiments. Error bars indicate standard deviations.

To investigate the growth of the gB 3A viruses, we performed single-step growth curve assays (Fig. 4A). Vero cells were infected with gB 3A-1, gB 3A-2, or GS3217 (WT) virus at 5 PFU/cell, and virus titers were determined at 6, 12, 18, and 24 hpi (Fig. 4A). A defect in gB 3A mutant virus replication was detected at 12, 18, and 24 hpi. By 24 hpi, the mutant virus titers were 3 log units lower than those of the GS3217 (WT) virus. To examine the replication of the gB 3A viruses at a lower multiplicity of infection (MOI), we performed a multistep growth curve assay (Fig. 4B). Vero cells were infected with the viruses at 0.01 PFU/cell, and virus titers were determined every 12 h until 48 hpi. At 12 hpi, the GS3217 (WT) virus titer had increased but the gB 3A virus titers had not. At 24 hpi, the gB 3A titers lagged behind the GS3217 (WT) virus titer by more than 5 log units (Fig. 4B). At 48 hpi, the gB 3A virus titers were increasing but had not reached the titer achieved by the GS3217 (WT) virus. Consistent with their small-plaque phenotype (Fig. 3) and delayed development of a CPE (Fig. 2), the gB 3A viruses demonstrated significant growth defects in both the single-step and multistep growth curves (Fig. 4).

### gB 3A viruses show slow penetration of cells.

The entry of HSV-1 into the cell begins with viral gB and/or gC attachment to cell surface HS proteoglycans. After binding, the virus is resistant to removal from the cell surface with phosphate-buffered saline (PBS) rinses but bound virions are sensitive to inactivation by a low-pH citrate buffer (21–25), Once fusion has occurred, the virions are no longer sensitive to inactivation by citrate buffer (21, 26, 27). This ability to differentiate between “bound viruses” and “fused viruses” by using low-pH inactivation allows penetration assays to be performed.

**FIG 5:**
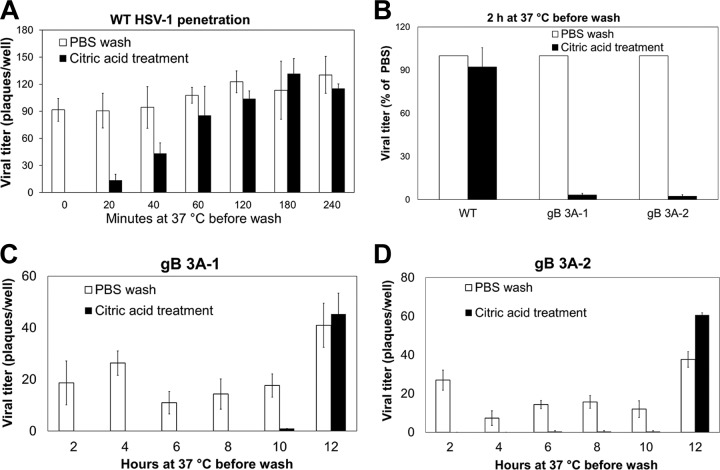
Penetration of cells by gB 3A viruses. (A) Penetration of cells by WT (GS3217) HSV-1. WT virus was added to Vero cell monolayers and incubated at 4°C for 1 h, and then the samples were shifted to 37°C for 20 to 240 min. At the times indicated, infected cells were rinsed with citrate buffer to inactivate extracellular virus or with PBS as a control. A methylcellulose overlay was added to the cells, and infection was quantified by a plaque assay. The data show the average number of plaques from one representative experiment performed in triplicate. (B) Penetration of cells by gB 3A and WT (GS3217) viruses. Vero monolayers in six-well plates were infected with WT, gB 3A-1, or gB 3A-2 virus. After 2 h at 37°C, cells were rinsed with either citric acid buffer or PBS (as a control). A methylcellulose overlay was added to the cells, and infection was quantified by a plaque assay. For each virus, the plaque counts after the citric acid wash are shown as a percentage of the plaque counts of that virus after the PBS rinse (the control). The data shown are from a representative experiment performed in triplicate. (C, D) Penetration of cells by gB 3A viruses. gB 3A-1 viruses (C) or gB 3A-2 viruses (D) were added to Vero cell monolayers in 24-well plates and incubated at 4°C for 1 h. The cells and viruses were shifted to 37°C for 0 to 12 h. At the times indicated, the infected cells were rinsed with citric acid buffer or PBS. A methylcellulose overlay was added to the cells, and the infection was quantified by a plaque assay. The data show the average number of plaques from one representative experiment performed in triplicate.

GS3217 (WT) or gB 3A HSV-1 (150 PFU) was allowed to adsorb to Vero cell monolayers for 1 h on ice. Cells were then incubated at 37°C for 0 to 4 h. At specific time points, cells were treated with citrate buffer to inactivate extracellular virus or with PBS as a control. After treatment, the cells were overlaid with methylcellulose medium and plaques were allowed to form. For G3217 (WT) virus, incubation at 37°C for 2 h prior to the citrate buffer treatment was sufficient to reach nearly maximal titers (Fig. 5A). Indeed, penetration of the G3217 (WT) virus was detectable after only 20 min at 37°C. In contrast, when the gB 3A viruses were allowed to adsorb to Vero cells for 1 h at 4°C and then allowed to enter the cells for 2 h at 37°C before citrate buffer inactivation, very few plaques formed (Fig. 5B). When the gB 3A viruses were rinsed with PBS instead of citrate buffer at 2 hpi, plaques formed as expected (Fig. 5B). These results demonstrate that at 37°C, the gB 3A viruses remain sensitive to low-pH inactivation longer than the G3217 (WT) virus.

To determine the time required for gB 3A virus penetration, the gB 3A viruses were allowed to bind to cells for 1 h on ice and then incubated at 37°C for up to 12 h prior to the citrate buffer treatment. Both gB 3A viruses did not show appreciable penetration of cells until 12 hpi (Fig. 5C and D). The control samples (gB 3A-infected cells treated with PBS instead of citrate buffer) showed plaques as expected. These results demonstrate a remarkable delay in the penetration kinetics of the gB 3A virus mutants (>10 h, compared to as little as 20 min for WT virus).

### gB 3A viruses do not exhibit a cell binding defect.

**FIG 6:**
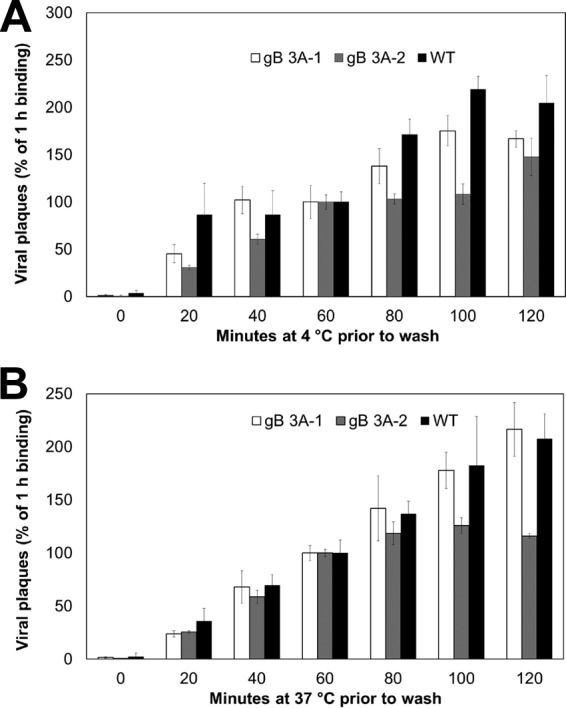
gB 3A virus binding to cells. WT (GS3217) and gB 3A viruses were diluted to 200 PFU/ml and added to Vero cells in a six-well plate at 4°C or 37°C. Samples were incubated for 0 to 120 min. At the time points indicated, the cells were rinsed twice with PBS to remove unbound virus and a methylcellulose overlay was added. Plaques were visualized with Giemsa stain at 3 days postinfection. For each virus, the level of binding achieved after 60 min of incubation was set to 100%. Normalization was performed independently for each temperature. The data show the plaque counts from one representative experiment performed in triplicate.

To examine whether poor binding might contribute to the slow growth kinetics of the gB 3A viruses, the cell binding kinetics of the gB 3A viruses and G3217 (WT) were compared. The gB 3A and G3217 (WT) viruses (200 PFU) were added to Vero cell monolayers, and the cells were incubated at 4°C or 37°C for up to 120 min. At 4°C, viruses can bind to but not fuse with the cells. At distinct time points, the cells were rinsed with PBS twice to remove unbound virus and methylcellulose medium was added. Plaques were counted after 3 days of incubation to determine the level of resistance to the PBS washes. The gB 3A and G3217 (WT) viruses showed similar plaque counts when incubated at 4°C (Fig. 6A) or 37°C (Fig. 6B) prior to the PBS washes. The plaque counts of all viruses increased as the incubation time prior to the PBS washes increased. These results suggest that the growth defect seen in the gB 3A viruses is not due to a severe cell binding defect in the gB 3A viruses.

### Complementation with hyperfusogenic gB rescues the small-plaque phenotype.

**FIG 7:**
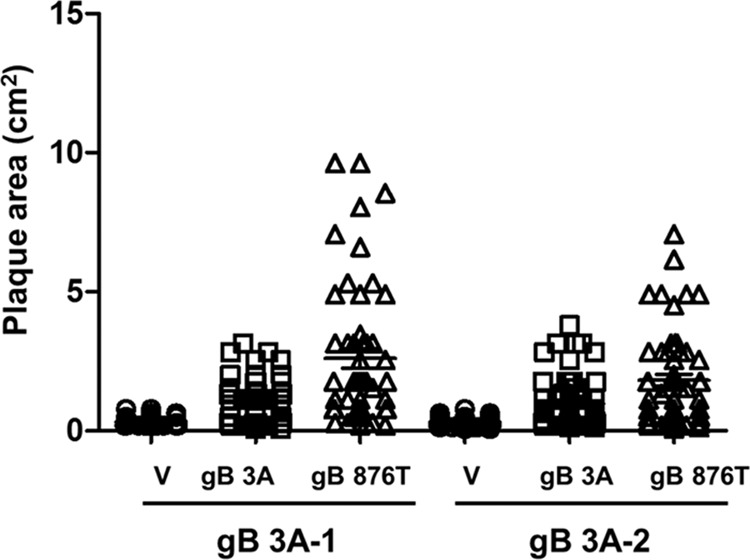
Restoration of plaque size by complementation with hyperfusogenic gB. Vero cells were transfected with empty-vector DNA (V) or a plasmid encoding gB 3A or gB 876T. After 24 h, the cells were infected with gB 3A-1 or gB 3A-2 virus for 2 h and overlaid with methylcellulose. Plaques were stained, and the radii of at least 40 randomly selected plaques from each virus were measured to calculate the plaque area. Each data point represents a single plaque.

To confirm that the small-plaque phenotype of the gB 3A virus maps to the gB 3A protein, we determined whether this phenotype could be rescued by complementing the virus with a hyperfusogenic form of gB (gB 876T) (20, 28) that has a truncation in its cytoplasmic tail. Vero cells were transfected overnight with a plasmid encoding gB 876T or gB 3A or with the vector. Transfected cells were infected with gB 3A-1 or gB 3A-2 virus, and plaques were imaged after 3 days. The plaque size on gB 876T-expressing cells was up to 10-fold larger than that on cells transfected with the vector (Fig. 7). These results indicate that expression of gB 876T partially rescues the gB 3A virus small-plaque phenotype, consistent with this phenotype mapping to the gB 3A protein. Interestingly, cells expressing gB 3A showed somewhat larger plaques than cells transfected with the vector, suggesting that greater levels of gB 3A expression may enhance virus spread.

### gB 3A virus cell penetration is partially rescued at higher temperature.

**FIG 8:**
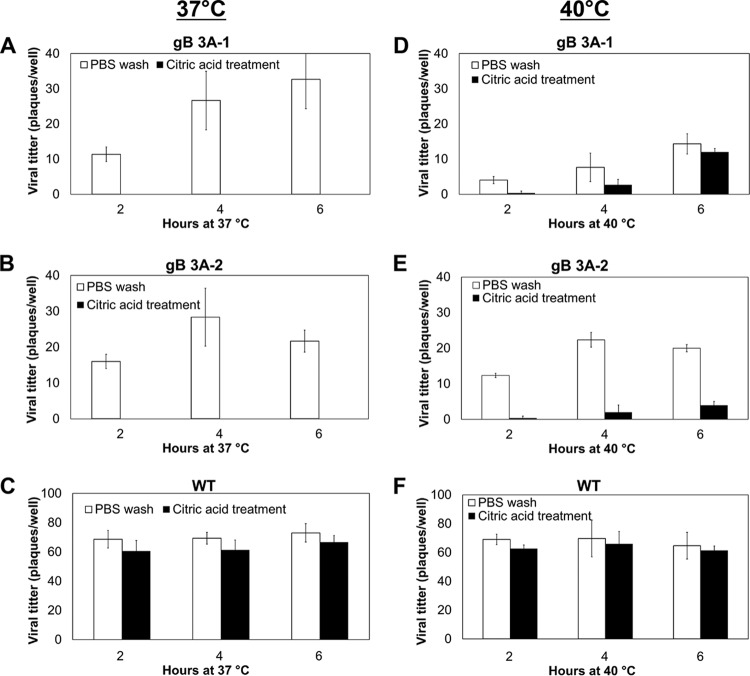
Penetration of Vero cells by gB 3A viruses at 40°C. gB 3A-1, gB 3A-2, or WT (GS3217) virus was added to Vero cell monolayers in 24-well plates and incubated at 4°C for 1 h. Samples then were shifted to 37°C (A to C) or 40°C (D to F) for 0 to 6 h. Cells were rinsed either with citric acid buffer to inactivate extracellular virus or with PBS as a control. A methylcellulose overlay was added to the cells, and infection was quantified by a plaque assay. The data show the average number of plaques from one representative experiment performed in triplicate.

If the triple alanine mutation in domain V of gB inhibits cell-cell fusion and virus cell penetration by favoring a prefusion conformation of the protein, the addition of heat might serve to rescue the fusion defect (29, 30). To determine whether an increase in temperature could promote the entry of the gB 3A viruses into cells, the penetration assay was performed at both 37°C (Fig. 8A to C) and 40°C (Fig. 8D to F). Viruses were allowed to penetrate cells for 2, 4, or 6 h before treatment with low-pH citrate buffer to inactivate extracellular virus or with PBS as a positive control. As expected, the citrate buffer treatment did not affect the entry of the G3217 (WT) compared with the PBS control because maximal penetration has occurred by 2 hpi (Fig. 8C). As previously seen, the gB 3A viruses failed to penetrate the cells after a 6-h incubation at 37°C (Fig. 8A and B); however, a small number of plaques were apparent when the gB 3A viruses were incubated with cells for 4 h at 40°C prior to citrate buffer treatment (Fig. 8D and E). These results suggest that the energy provided by the increased temperature may overcome the penetration defect in the gB 3A virus, potentially by promoting the prefusion-to-postfusion conformational change in gB.

### Excess gH/gL does not restore the fusion activity of the gB 3A mutant protein.

**FIG 9:**
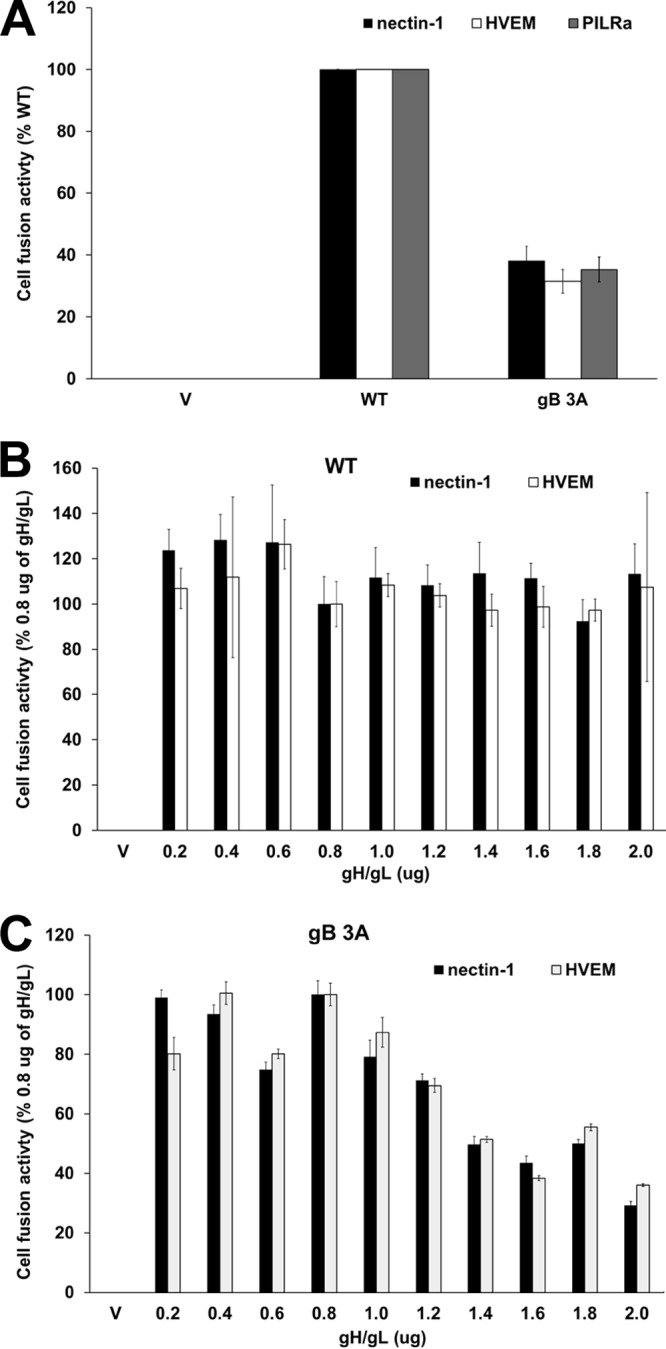
gB 3A-mediated cell-cell fusion. (A) Cell-cell fusion activity of the WT gB and gB 3A proteins. Target CHO cells were transfected with a reporter plasmid encoding luciferase under the control of the T7 promoter along with a plasmid encoding the PILRα, HVEM, or nectin-1 receptor. Transfected cells were cocultured with effector CHO cells that had been transfected with plasmids encoding T7 polymerase, gD, gH, gL, and either WT gB or gB 3A. After coincubation overnight, luciferase activity was measured as an indication of cell-cell fusion. Background signals (effector cells transfected with the vector only) were subtracted, and the data were normalized to the fusion activity observed with WT gB. Data were normalized separately for each receptor. Each bar shows the mean and standard deviation of three independent experiments. (B, C) Impact of gH/gL levels on fusion mediated by WT gB (B) or gB 3A (C). Target CHO cells were transfected with a reporter plasmid encoding luciferase under the control of the T7 promoter and a plasmid encoding either HVEM or nectin-1. Effector CHO cells were transfected separately with plasmids encoding T7 polymerase, gD, gH, gL, and gB (B) or gB 3A (C). Increasing amounts of gH and gL plasmid DNA were added to the effector cell transfections. Target cells were cocultured with effector cells overnight, and luciferase activity was measured as an indication of cell-cell fusion. Background signals (effector cells transfected with the vector only) were subtracted, and data were normalized to the level of fusion achieved by WT gB in cells transfected with 0.8 µg each of the gH and gL plasmids (standard gH/gL levels). Fusion with each receptor was normalized independently. V denotes empty-vector DNA transfected into the effector cells. Each bar shows the mean and standard deviation of three independent determinations.

We previously demonstrated that the gB 3A mutant protein exhibited reduced fusion in a cell-cell fusion assay using the HSV receptors nectin-1 and HVEM (20). A quantitative cell-cell fusion assay was used to confirm this reduced fusion capacity and to test gB 3A fusion by using the PILRα receptor, an entry receptor that binds to gB. Fusion was reduced with all of the HSV receptors tested (Fig. 9A). If the gB 3A mutations favor the prefusion conformation of gB as we hypothesize, reduced fusion with all HSV receptors is expected.

The current model of HSV entry proposes that the gH/gL heterodimer conveys a signal to gB to trigger fusion (31). To explore whether a defect in the gB-gH/gL interaction might contribute to the penetration defect observed in the gB 3A viruses, a cell-cell fusion assay was used. This assay permits the titration of different levels of gH/gL DNA, which results in different levels of protein expression. Expression of higher levels of gH/gL may compensate for a weak interaction between gB 3A and gH/gL. When cells were cotransfected with gD, gH, gL, and gB 3A, increasing the amounts of gH and gL DNA transfected did not enhance cell-cell fusion (Fig. 9C). In fact, greater levels of gH/gL DNA impaired the fusion mediated by gB 3A. These results do not exclude the possibility that the gB 3A mutant has a defect in gH/gL interaction; however, the results do not support the hypothesis either.

## DISCUSSION

Previously, mutations in the C-terminal region of the gB ectodomain (the domain V arm) demonstrated that this region of gB is important for fusion function (20). Triple alanine mutations (I671A, H681A, and F683A) in this gB arm region resulted in decreased cell-cell and virus-cell fusion, despite WT levels of cell surface expression and monoclonal antibody (MAb) reactivity (20). To investigate the effect of the triple alanine mutations on virus entry, we constructed HSV-1 gB 3A viruses carrying these mutations by using a BAC (32, 33) and characterized the entry of these viruses into cells.

The gB 3A viruses formed plaques on Vero cells that were 200-fold smaller than WT HSV-1 plaques (Fig. 3A). Growing the gB 3A virus on Vero cells expressing WT gB (VgB cells) partially rescued the small-plaque phenotype (Fig. 3B), suggesting that the mutations in gB 3A are responsible for the defect in plaque size. Similarly, complementation with hyperfusogenic gB 876T also restored plaque size (Fig. 7). Single-step and multistep growth curves demonstrated a defect in the growth kinetics of the gB 3A viruses (Fig. 4). Correspondingly, gB 3A viruses took longer than the WT virus to spread through a culture of Vero cells (Fig. 2). gB 3A protein displays a reduced fusion capacity (Fig. 9A) that may explain the small-plaque phenotype and delayed growth of the gB 3A viruses.

The growth defects may be due to impaired and/or delayed cell-to-cell spread of gB 3A virus. In addition, defects in gB 3A egress also may contribute to its small-plaque phenotype and delayed growth. gB has been reported to function during virus egress and during both de-envelopment at the outer nuclear membrane and secondary envelopment (34, 35). When the gB fusion loops were mutated, de-envelopment was impaired. The reduced fusion capacity of gB 3A may hinder de-envelopment similarly.

By using a low pH to inactivate extracellular virus at defined time points, we demonstrated that the gB 3A viruses have remarkably delayed penetration of cells. Penetration of Vero cells by WT HSV-1 was detectable after 20 min at 37°C and reached a maximum after 2 h at 37°C (Fig. 5A). In contrast, the gB 3A viruses did not penetrate the cells until 10 to 12 h at 37°C (Fig. 5C and D). HSV entry into Vero cells occurs at the cell surface (36, 37), and thus, the delayed penetration kinetics of gB 3A may be due to a defect in the ability of gB 3A to mediate fusion, rather than an effect on endocytosis.

HSV-1 gB and gC bind to cell surface HS proteoglycans, and viruses with these glycoproteins deleted are impaired in the ability to attach to cells (21, 26). HS binding was mapped to a lysine-rich region of gB (residues 68 to 76) (38). Mutation of this lysine-rich region disrupts HS binding, and the mutant virus is impaired but remains infectious, indicating that gB binding to HS is not essential for infection (38). The gB 3A mutations are distant from the HS binding site, so these mutations were not expected to affect HS binding. When cell binding was examined by using a washout assay, the gB 3A and WT viruses demonstrated similar degrees of retention on the cell (Fig. 6), suggesting that the gB 3A mutations did not impact cell binding.

In addition to mediating membrane fusion and binding to HS, gB is proposed to interact with gH/gL. In the current model of entry, gD binding to an entry receptor relays a signal to gH/gL that is transmitted to gB to trigger fusion (31). To explore whether the defects in gB 3A function are due to a deficiency in its gH/gL interaction, we used a cell-cell fusion assay that allowed us to titrate increasing levels of gH/gL DNA (Fig. 9). If the gB 3A fusion defect is due to a reduced capacity to interact with the fusion regulator gH/gL, higher levels of gH/gL expression might compensate for the defect. Transfection with high levels of gH/gL DNA did not enhance fusion mediated by gB 3A, and thus, the hypothesis that gB 3A has impaired an interaction with gH/gL was not supported.

The refolding of a fusion protein from a prefusion to a postfusion conformation requires energy. For herpesviruses, an interaction with gH/gL is proposed to destabilize the prefusion conformation of gB to trigger the conformational change that mediates fusion. Heat has been used previously as a surrogate trigger for several class I and III fusion proteins to transition from a prefusion to a postfusion conformation (29, 39–45). To explore whether the defects in gB 3A fusion could be overcome by heat, the viral penetration rate was examined at an elevated temperature. At 37°C, penetration by the gB 3A viruses required more than 10 h of incubation with cells (Fig. 5); however, penetration was detectable after only 4 h at 40°C (Fig. 8). Although heat may impact multiple stages of the HSV-1 infectious cycle, WT virus penetration was unaffected by the 40°C incubation. The absence of a nonspecific enhancement of WT virus penetration at 40°C suggests that heat partially rescued the cell penetration of the gB 3A virus by affecting the gB 3A protein.

**FIG 10:**
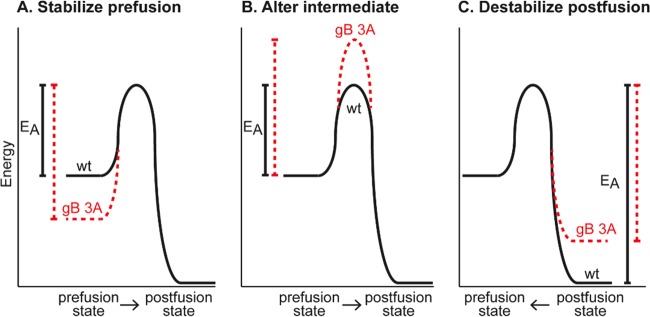
Hypothetical models of gB refolding energetics. Although gB refolding likely progresses through several intermediate conformations, only the prefusion and postfusion conformation energy states are depicted. The hypothetical impact of the gB 3A mutations on these states is shown in red. Comparisons of energy of activation (E_A_) are shown as vertical lines. (A) The 3A mutations may stabilize the gB prefusion conformation, thereby increasing the E_A_ required to trigger gB and reducing gB fusion capacity. (B) The 3A mutations may increase the E_A_ required to achieve an intermediate conformation of gB, such as a prehairpin intermediate, thereby reducing the gB fusion capacity. (C) By decreasing the affinity of the arm for the coil, the 3A mutations may destabilize the postfusion gB, thereby reducing the E_A_ required to move from a postfusion to a prefusion conformation. At equilibrium, this would increase the proportion of the gB population in a prefusion state.

The gB 3A mutations were designed to reduce interactions between the domain V arm and the helical central coiled coil of gB (20), on the basis of the postfusion structure of HSV-1 gB (12) (Fig. 1A). Because of the similarity of the gB coil-arm complex to the six-helix bundle of class I fusion proteins, we hypothesized that the gB coil-arm interactions are important for the transition from a prefusion to a postfusion conformation and that gB 3A mutations would alter the energetics of gB refolding (Fig. 10). The slow-entry phenotype of the gB 3A viruses and the ability of heat to rescue their cell penetration support our hypothesis that interaction between the arm and coil regions of gB is an important step in the refolding of gB (20).

Upon triggering, the gB 3A mutant may fold to a postfusion form that is inefficient at fusing membranes. Alternatively, the gB 3A mutations may alter the activation energy barriers that regulate the transitions between the prefusion, intermediate, and postfusion conformations. Infrequent or slow conversion of gB 3A from a prefusion to a postfusion conformation may account for the slow-penetration phenotype of the gB 3A virus. If the gB 3A mutations increase the activation energy required to trigger gB by either stabilizing the prefusion form (Fig. 10A) or destabilizing an intermediate conformation (Fig. 10B), the rate of fusion would be decreased.

Destabilizing the postfusion form of gB also could impact fusion by altering the thermodynamic equilibrium between the prefusion and postfusion forms in the gB population (Fig. 10C). Although reversibility of the gB conformational change has not been demonstrated, the refolding of rhabdovirus fusion protein G (another class III fusion protein) from a prefusion to a postfusion conformation is reversible (46–48). If the prefusion and postfusion conformations of rhabdovirus G exist in equilibrium, mutations that destabilize the postfusion form could decrease fusion capacity by shifting the equilibrium toward the prefusion state. The gB 3A mutations may destabilize the postfusion gB conformation by decreasing the affinity of the arm for the coil and thereby push the equilibrium within the gB population toward the prefusion state.

The gB 3A mutations may provide tools to study the prefusion form of gB, potentially by contributing to the stabilization of prefusion gB in future crystallization trials. Future experiments will select for second-site revertant mutations to identify potential interaction sites within gB and to investigate how other viral proteins regulate gB fusion activity.

## MATERIALS AND METHODS

### Cells and antibodies.

Chinese hamster ovary (CHO-K1; American Type Culture Collection [ATCC], Manassas, VA) cells were grown in Ham’s F-12 medium supplemented with 10% fetal bovine serum (FBS; Thermo Fisher Scientific), Vero cells (ATCC), Vero-B24 cells (VgB; kindly provided by Patricia Spear, Northwestern University) stably expressing HSV-1 gB (21), and Vero-Cre cells (kindly provided by Gregory Smith, Northwestern University) expressing Cre recombinase were grown in Dulbecco modified Eagle medium (DMEM) supplemented with 10% FBS, penicillin, and streptomycin.

The antibodies used in this study included rabbit anti-gB polyclonal antibody (PAb) serum R74 (kindly provided by Patricia Spear, Northwestern University) (26) and the anti-VP5 MAb (East Coast Bio).

### Plasmids.

Plasmids expressing HSV-1 (KOS strain) gB (pPEP98), gD (pPEP99), gH (pPEP100), and gL (pPEP101) (kindly provided by Patricia Spear, Northwestern University) were previously described (49), as were plasmids expressing human nectin-1 (pBG38; kindly provided by Patricia Spear, Northwestern University) (6), HVEM (pBEC10; kindly provided by Patricia Spear, Northwestern University) (4), and human PILRα (pQF003) (50). pGS1439, a plasmid containing the Kan^r^-encoding gene, was kindly provided by Gregory Smith, Northwestern University. pSG5-HSVgB-I671A/H681A/F683A contains HSV-1 gB carrying three alanine mutations in domain V (3A here) (20).

### Virus construction.

To construct the HSV-1 gB 3A viruses, we first created a gB-null virus by using BAC pGS3217 (kindly provided by Gregory Smith, Northwestern University). GS3217 is an HSV-1 F strain that carries the red fluorescent protein (RFP) tdTomato reporter gene with a nuclear localization signal under the control of a cytomegalovirus (CMV) promoter. GS3217 was derived from a BAC kindly provided by Yasushi Kawaguchi, University of Tokyo (51). The CMV>NLS-tdTomato>pA cassette is inserted after the start codon of US5 (gJ) and introduces an in-frame stop codon. First, the Kan^r^-encoding gene was amplified from pGS1439 by PCR with primers TGCACATGCGGTTTAACACCCGTGGTTTTTATTTACAACAAACCCCCCGGGCGGGACTACGGGG**AGGATGACGACGATAAGTAGGG** and GCCCCCAGGCTACCTGACGGGGGGCACGACGGGCCCCCGTAGTCCCGCCCGGGGGGTTTGTTGT**CAACCAATTAACCAATTCTGAT**, which consist of UL27 (gB) flanking sequence and Kan^r^ homology (in bold). By using a two-step red-mediated recombination strategy (32, 33), this PCR product was recombined into pGS3217 in place of UL27 (gB) to delete WT UL27 and then the Kan^r^ cassette was recombined out to generate gB-null BAC pQF282 (Fig. 1B and C).

To add the mutant gB 3A-encoding gene to gB-null BAC pQF282, the Kan^r^-encoding gene was amplified from pGS1439 by PCR with primers CGTTCC**ACCGGT**ACGGGACGACGGTAAACTGCATCGTCGAGGAGGTGGACGCGCGCTCGGTGTAGGATGACGACGATAAGTAGGGA and TCCCGT**ACCGGT**CAACCAATTAACCAATTCTGAT, which consist of UL27 (gB) sequence (including an AgeI restriction site [in bold]) and Kan^r^ homology (underlined). This PCR product was digested with AgeI and ligated into AgeI-digested pSG5-HSVgB-I671A/H681A/F683A (20) to generate pQF270. pSG5-HSVgB-I671A/H681A/F683A is a plasmid that carries the gB 3A mutant gene and contains a unique AgeI site located in the middle of the gB-encoding gene. The gB 3A-encoding gene containing a Kan^r^-encoding gene insert was amplified from pQF270 by PCR with primers ACAAACCAAAAGATGCACATGCGGTTTAACACCCGTGGTTTTTATTTACAACAAACCCCCCGTCACAGGTCGTCCTCGTCGGCGTC and CCTCCAGCACCTCGCCCCCAGGCTACCTGACGGGGGGCACGACGGGCCCCCGTAGTCCCGCCATGCACCAGGGCGCCCCCTCGTGG. By two-step red-mediated recombination, this PCR product was recombined into gB-null BAC pQF282 and then the Kan^r^ cassette was recombined out to generate BAC pQF297, which carries the gB 3A mutant gene in place of WT gB (Fig. 1D). At each step of the BAC constructions, the intermediate BACs with Kan^r^-encoding gene insertions and the final BACs were confirmed by at least four restriction enzyme digestions.

BAC pQF297 was transfected into Vero cells expressing Cre recombinase by using Lipofectamine 2000 (Invitrogen, Carlsbad, CA). The transfected cells were harvested 3 weeks after transfection, sonicated, and subsequently passaged on Vero cells to generate HSV-1 gB 3A stocks. gB 3A-1 and gB 3A-2 viruses were isolated from two separate transfections of pQF297, and both contain the same triple alanine mutation in gB, as confirmed by sequencing. WT GS3217 virus was harvested at 6 to 7 days posttransfection. Viral DNA was sequenced by the Northwestern Genomic core facility.

### Western blot assays.

Western blot assays were performed to examine the expression of HSV-1 gB (50). Viruses harvested from Vero cells infected at an MOI of 0.01 were lysed in 200 μl of lysis buffer (25 mM Tris-HCl [pH 7.4], 150 mM NaCl, 5 mM EDTA, 10 mM NaF, 1 mM Na_3_VO_3_, 1% Nonidet P-40) containing protease inhibitors (Roche Diagnostics, Indianapolis, IN). Proteins were boiled for 5 min under reducing conditions, separated by SDS-PAGE on 4 to 20% gels, and transferred to nitrocellulose. Blots were probed with anti-gB PAb R74 at a 1:10,000 dilution for 1 h at room temperature, followed by IRDye 800CW goat anti-rabbit IgG (LI-COR, Lincoln, NE) at 1:10,000. The bands were visualized by Odyssey imaging (LI-COR). Incorporation of gB 3A into the virion was examined as previously reported (52). Briefly, medium from infected Vero cells containing extracellular virus was subjected to low-speed centrifugation to pellet cells and debris. The supernatant was centrifuged at 48,000 × *g* for 1 h at 4°C over a 10% sucrose cushion. The pellet was dissolved in sample buffer and separated by SDS-PAGE on a 4 to 20% gel under reducing conditions. Western blot analysis was performed with R74 at a 1:10,000 dilution and anti-VP5 MAb (East Coast Bio) at a 1:2,000 dilution, followed by the addition of IRDye 800CW goat anti-rabbit IgG (LI-COR, Lincoln, NE) and IRDye 680LT donkey anti-mouse antibody at 1:10,000 (LI-COR, Lincoln, NE).

### Microscopy and determination of plaque size.

Fluorescence microscopy of RFP expression in infected Vero cells was performed with an EVOS Cell Imaging System (AMG, Fisher Scientific). Plaques of both WT and gB 3A viruses were visualized by Giemsa (Sigma-Aldrich) staining after 3 days of incubation and then imaged by transmitted-light microscopy with an EVOS Cell Imaging System. Plaques size was calculated by using the EVOS Cell Imaging System. Randomly selected plaques were enlarged on a computer screen, and the average plaque radius was calculated from two independent measurements of at least 20 plaques. By using this average radius, the plaque area and the ratio of the plaque sizes of mutant and WT viruses were determined.

### Single-step growth curve analysis.

To analyze viral replication kinetics, a single-step growth curve was determined as previously described (53, 54). Briefly, Vero cells in six-well plates were infected with the HSV-1 gB 3A-1, HSV-1 3A-2, or GS3217 (WT) virus at 5 PFU/cell for 1 h. The inoculum was removed, and the cells were rinsed twice with PBS and then incubated at 37°C (time zero, binding time not included). After 6, 12, 18, 24, and 48 h of incubation, cells were harvested and freeze-thawed at −40°C three times and virus titers were determined by plaque assay on Vero cells.

### Multiple-step growth curve analysis.

Multiple-step growth curves were determined as previously described (55). Vero cell monolayers in six-well plates were infected with HSV-1 gB 3A or GS3217 (WT) virus at 0.01 PFU/cell for 1 h. The inoculum was removed, and the cells were rinsed twice with PBS and then incubated at 37°C (time zero). At 0, 12, 24, 36, and 48 h of incubation, cells were harvested and freeze-thawed at −40°C three times and virus titers were determined by plaque assay on Vero cells.

### Virus cell penetration kinetics.

To assay the cell entry of the HSV-1 gB 3A viruses, a cell penetration experiment was performed as previously described (56). GS3217 (WT) virus or HSV-1 gB 3A viruses were diluted to 150 PFU/ml in PBS and allowed to adsorb to Vero cell monolayers in six-well plates for 1 h on ice, Cells were washed three times with cold PBS, and then 1 ml/well of medium prewarmed to 37°C or 40°C was added (time zero). Cells were incubated at 37°C or 40°C for up to 12 h. At the time points indicated, cells were treated for 1 min with citrate buffer (135 mM NaCl, 10 mM KCl, 40 mM citric acid, pH 3.0) or with PBS as a control. The cells were carefully rinsed five times with PBS. The monolayers were overlaid with methylcellulose medium (0.5% methylcellulose in DMEM with 1% heat-inactivated serum [Sigma-Aldrich]) and incubated at 37°C for 3 days for HSV-1 3A viruses and WT virus. Plaques were visualized by Giemsa staining and counted under light microcopy.

### Virus binding assay.

HSV-1 gB 3A or GS3217 (WT) virus was diluted to 200 PFU/ml in PBS with 1% heat-inactivated calf serum and 1% glucose, and 1 ml was added to Vero cell monolayers in six-well plates at 4°C or 37°C. Plates were incubated for 0, 20, 40, 60, 80, 100, or 120 min. The cells were rinsed twice with PBS, overlaid with methylcellulose (Sigma-Aldrich) medium, and incubated at 37°C for 3 days. Plaques were detected by Giemsa staining.

### Plaque size determination after gB mutant complementation.

Vero cells in six-well plates were transfected with 1,500 ng of the vector or a plasmid encoding gB 3A or gB 876T. After 24 h, cells were infected with about 200 PFU of gB 3A-1 or gB 3A-2 virus for 2 h, followed by methylcellulose overlay. After 3 days of incubation, plaques were visualized by Giemsa staining and imaged by transmitted-light microscopy with an EVOS Cell Imaging System. The radii of at least 40 randomly selected plaques were measured, and the plaque area was calculated on the basis of the radii.

### Cell-cell fusion assay.

The cell-cell fusion assay was performed as previously described (49). Briefly, CHO-K1 cells were seeded into six-well plates and incubated overnight. One set of cells (effectors) was transfected with plasmids encoding T7 RNA polymerase, gD, gH, and gL, as well as WT gB, mutant gB, or empty vector, using 5 µl of Lipofectamine 2000 (Invitrogen) and 400 ng of each plasmid. In some experiments, the total amount of gH and gL plasmid DNA transfected was titrated from 200 ng/well to 2 µg/well. A second set of cells (targets) was transfected with 400 ng of a plasmid carrying the firefly luciferase-encoding gene under the control of the T7 promoter and 1.5 µg of the empty vector or a plasmid encoding PILRα, HVEM, or nectin-1 with 5 µl of Lipofectamine 2000. After 6 h of transfection, the cells were detached with Versene and resuspended in 1.5 ml of F12 medium supplemented with 10% FBS. Effector and target cells were mixed at a 1:1 ratio and replated in 96-well plates for 18 h. Luciferase activity was quantified with a luciferase reporter assay system (Promega) in a Wallac-Victor luminometer (PerkinElmer).

## References

[1] Connolly SA, Jackson JO, Jardetzky TS, Longnecker R 2011 Fusing structure and function: a structural view of the herpesvirus entry machinery. Nat Rev Microbiol 9:369–381. doi:10.1038/nrmicro2548. 21478902 PMC3242325

[2] Eisenberg RJ, Atanasiu D, Cairns TM, Gallagher JR, Krummenacher C, Cohen GH 2012 Herpesvirus fusion and entry: a story with many characters. Viruses 4:800–832. doi:10.3390/v4050800. 22754650 PMC3386629

[3] Stampfer SD, Heldwein EE 2013 Stuck in the middle: structural insights into the role of the gH/gL heterodimer in herpesvirus entry. Curr Opin Virol 3:13–19. doi:10.1016/j.coviro.2012.10.005. 23107819 PMC3562363

[4] Montgomery RI, Warner MS, Lum BJ, Spear PG 1996 Herpes simplex virus-1 entry into cells mediated by a novel member of the TNF/NGF receptor family. Cell 87:427–436. doi:10.1016/S0092-8674(00)81363-X. 8898196

[5] Cocchi F, Menotti L, Mirandola P, Lopez M, Campadelli-Fiume G 1998 The ectodomain of a novel member of the immunoglobulin subfamily related to the poliovirus receptor has the attributes of a bona fide receptor for herpes simplex virus types 1 and 2 in human cells. J Virol 72:9992–10002. 9811737 10.1128/jvi.72.12.9992-10002.1998 PMC110516

[6] Geraghty RJ, Krummenacher C, Cohen GH, Eisenberg RJ, Spear PG 1998 Entry of alphaherpesviruses mediated by poliovirus receptor-related protein 1 and poliovirus receptor. Science 280:1618–1620. doi:10.1126/science.280.5369.1618. 9616127

[7] Lopez M, Cocchi F, Menotti L, Avitabile E, Dubreuil P, Campadelli-Fiume G 2000 Nectin2α (PRR2α or HveB) and nectin2δ are low-efficiency mediators for entry of herpes simplex virus mutants carrying the Leu25Pro substitution in glycoprotein D. J Virol 74:1267–1274. doi:10.1128/JVI.74.3.1267-1274.2000. 10627537 PMC111461

[8] Warner MS, Geraghty RJ, Martinez WM, Montgomery RI, Whitbeck JC, Xu R, Eisenberg RJ, Cohen GH, Spear PG 1998 A cell surface protein with herpesvirus entry activity (HveB) confers susceptibility to infection by mutants of herpes simplex virus type 1, herpes simplex virus type 2 and pseudorabies virus. Virology 246:179–189. doi:10.1006/viro.1998.9218. 9657005

[9] Shukla D, Liu J, Blaiklock P, Shworak NW, Bai X, Esko JD, Cohen GH, Eisenberg RJ, Rosenberg RD, Spear PG 1999 A novel role for 3-*O*-sulfated heparan sulfate in herpes simplex virus 1 entry. Cell 99:13–22. doi:10.1016/S0092-8674(00)80058-6. 10520990

[10] Shukla D, Spear PG 2001 Herpesviruses and heparan sulfate: an intimate relationship in aid of viral entry. J Clin Invest 108:503–510. doi:10.1172/JCI13799. 11518721 PMC209412

[11] Satoh T, Arii J, Suenaga T, Wang J, Kogure A, Uehori J, Arase N, Shiratori I, Tanaka S, Kawaguchi Y, Spear PG, Lanier LL, Arase H 2008 PILRα is a herpes simplex virus-1 entry co-receptor that associates with glycoprotein B. Cell 132:935–944. 18358807 10.1016/j.cell.2008.01.043 PMC2394663

[12] Heldwein EE, Lou H, Bender FC, Cohen GH, Eisenberg RJ, Harrison SC 2006 Crystal structure of glycoprotein B from herpes simplex virus 1. Science 313:217–220. doi:10.1126/science.1126548. 16840698

[13] Cooper RS, Heldwein EE 2015 Herpesvirus gB: a finely tuned fusion machine. Viruses 7:6552–6569. doi:10.3390/v7122957. 26690469 PMC4690880

[14] Backovic M, Longnecker R, Jardetzky TS 2009 Structure of the Epstein-Barr virus glycoprotein B. Proc Natl Acad Sci U S A 106:2880–2885. doi:10.1073/pnas.0810530106. 19196955 PMC2650359

[15] Burke HG, Heldwein EE 2015 Correction: crystal structure of the human cytomegalovirus glycoprotein B. PLoS Pathog 11:e1005300. doi:10.1371/journal.ppat.1005300. 26484870 PMC4617298

[16] Chandramouli S, Ciferri C, Nikitin PA, Caló S, Gerrein R, Balabanis K, Monroe J, Hebner C, Lilja AE, Settembre EC, Carfi A 2015 Structure of HCMV glycoprotein B in the postfusion conformation bound to a neutralizing human antibody. Nat Commun 6:8176. doi:10.1038/ncomms9176. 26365435 PMC4579600

[17] Vitu E, Sharma S, Stampfer SD, Heldwein EE 2013 Extensive mutagenesis of the HSV-1 gB ectodomain reveals remarkable stability of its postfusion form. J Mol Biol 425:2056–2071. doi:10.1016/j.jmb.2013.03.001. 23500487 PMC3655159

[18] White JM, Delos SE, Brecher M, Schornberg K 2008 Structures and mechanisms of viral membrane fusion proteins: multiple variations on a common theme. Crit Rev Biochem Mol Biol 43:189–219. doi:10.1080/10409230802058320. 18568847 PMC2649671

[19] Zeev-Ben-Mordehai T, Vasishtan D, Hernández Durán A, Vollmer B, White P, Prasad Pandurangan A, Siebert CA, Topf M, Grünewald K 2016 Two distinct trimeric conformations of natively membrane-anchored full-length herpes simplex virus 1 glycoprotein B. Proc Natl Acad Sci U S A 113:4176–4181. doi:10.1073/pnas.1523234113. 27035968 PMC4839410

[20] Connolly SA, Longnecker R 2012 Residues within the C-terminal arm of the herpes simplex virus 1 glycoprotein B ectodomain contribute to its refolding during the fusion step of virus entry. J Virol 86:6386–6393. doi:10.1128/JVI.00104-12. 22491468 PMC3393567

[21] Herold BC, WuDunn D, Soltys N, Spear PG 1991 Glycoprotein C of herpes simplex virus type 1 plays a principal role in the adsorption of virus to cells and in infectivity. J Virol 65:1090–1098. 1847438 10.1128/jvi.65.3.1090-1098.1991 PMC239874

[22] McClain DS, Fuller AO 1994 Cell-specific kinetics and efficiency of herpes simplex virus type 1 entry are determined by two distinct phases of attachment. Virology 198:690–702. doi:10.1006/viro.1994.1081. 8291250

[23] Huang AS, Wagner RR 1964 Penetration of herpes simplex virus into human epidermoid cells. Proc Soc Exp Biol Med 116:863–869. doi:10.3181/00379727-116-29392. 14230373

[24] Highlander SL, Cai WH, Person S, Levine M, Glorioso JC 1988 Monoclonal antibodies define a domain on herpes simplex virus glycoprotein B involved in virus penetration. J Virol 62:1881–1888. 2452895 10.1128/jvi.62.6.1881-1888.1988 PMC253270

[25] Highlander SL, Sutherland SL, Gage PJ, Johnson DC, Levine M, Glorioso JC 1987 Neutralizing monoclonal antibodies specific for herpes simplex virus glycoprotein D inhibit virus penetration. J Virol 61:3356–3364. 2444713 10.1128/jvi.61.11.3356-3364.1987 PMC255929

[26] Herold BC, Visalli RJ, Susmarski N, Brandt CR, Spear PG 1994 Glycoprotein C-independent binding of herpes simplex virus to cells requires cell surface heparan sulphate and glycoprotein B. J Gen Virol 75:1211–1222. doi:10.1099/0022-1317-75-6-1211. 8207388

[27] Shieh MT, WuDunn D, Montgomery RI, Esko JD, Spear PG 1992 Cell surface receptors for herpes simplex virus are heparan sulfate proteoglycans. J Cell Biol 116:1273–1281. doi:10.1083/jcb.116.5.1273. 1310996 PMC2289355

[28] Chowdary TK, Heldwein EE 2010 Syncytial phenotype of C-terminally truncated herpes simplex virus type 1 gB is associated with diminished membrane interactions. J Virol 84:4923–4935. doi:10.1128/JVI.00206-10. 20200237 PMC2863819

[29] Chesnokova LS, Ahuja MK, Hutt-Fletcher LM 2014 Epstein-Barr virus glycoprotein gB and gHgL can mediate fusion and entry in trans, and heat can act as a partial surrogate for gHgL and trigger a conformational change in gB. J Virol 88:12193–12201. doi:10.1128/JVI.01597-14. 25142593 PMC4248918

[30] Paterson RG, Russell CJ, Lamb RA 2000 Fusion protein of the paramyxovirus SV5: destabilizing and stabilizing mutants of fusion activation. Virology 270:17–30. doi:10.1006/viro.2000.0267. 10772976

[31] Atanasiu D, Saw WT, Cohen GH, Eisenberg RJ 2010 Cascade of events governing cell-cell fusion induced by herpes simplex virus glycoproteins gD, gH/gL, and gB. J Virol 84:12292–12299. doi:10.1128/JVI.01700-10. 20861251 PMC2976417

[32] Tischer BK, Smith GA, Osterrieder N 2010 En passant mutagenesis: a two step markerless red recombination system. Methods Mol Biol 634:421–430. doi:10.1007/978-1-60761-652-8_30. 20677001

[33] Tischer BK, von Einem J, Kaufer B, Osterrieder N 2006 Two-step red-mediated recombination for versatile high-efficiency markerless DNA manipulation in Escherichia coli. Biotechniques 40:191–197. 16526409 10.2144/000112096

[34] Johnson DC, Wisner TW, Wright CC 2011 Herpes simplex virus glycoproteins gB and gD function in a redundant fashion to promote secondary envelopment. J Virol 85:4910–4926. doi:10.1128/JVI.00011-11. 21411539 PMC3126161

[35] Wright CC, Wisner TW, Hannah BP, Eisenberg RJ, Cohen GH, Johnson DC 2009 Fusion between perinuclear virions and the outer nuclear membrane requires the fusogenic activity of herpes simplex virus gB. J Virol 83:11847–11856. doi:10.1128/JVI.01397-09. 19759132 PMC2772685

[36] Nicola AV, McEvoy AM, Straus SE 2003 Roles for endocytosis and low pH in herpes simplex virus entry into HeLa and Chinese hamster ovary cells. J Virol 77:5324–5332. doi:10.1128/JVI.77.9.5324-5332.2003. 12692234 PMC153978

[37] Spear PG, Longnecker R 2003 Herpesvirus entry: an update. J Virol 77:10179–10185. doi:10.1128/JVI.77.19.10179-10185.2003. 12970403 PMC228481

[38] Laquerre S, Argnani R, Anderson DB, Zucchini S, Manservigi R, Glorioso JC 1998 Heparan sulfate proteoglycan binding by herpes simplex virus type 1 glycoproteins B and C, which differ in their contributions to virus attachment, penetration, and cell-to-cell spread. J Virol 72:6119–6130. 9621076 10.1128/jvi.72.7.6119-6130.1998 PMC110418

[39] Carr CM, Chaudhry C, Kim PS 1997 Influenza hemagglutinin is spring-loaded by a metastable native conformation. Proc Natl Acad Sci U S A 94:14306–14313. doi:10.1073/pnas.94.26.14306. 9405608 PMC24954

[40] Ruigrok RWH, Martin SR, Wharton SA, Skehel JJ, Bayley PM, Wiley DC 1986 Conformational changes in the hemagglutinin of influenza virus which accompany heat-induced fusion of virus with liposomes. Virology 155:484–497. doi:10.1016/0042-6822(86)90210-2. 3788061

[41] Ader N, Brindley M, Avila M, Örvell C, Horvat B, Hiltensperger G, Schneider-Schaulies J, Vandevelde M, Zurbriggen A, Plemper RK, Plattet P 2013 Mechanism for active membrane fusion triggering by morbillivirus attachment protein. J Virol 87:314–326. doi:10.1128/JVI.01826-12. 23077316 PMC3536382

[42] Connolly SA, Leser GP, Yin HS, Jardetzky TS, Lamb RA 2006 Refolding of a paramyxovirus F protein from prefusion to postfusion conformations observed by liposome binding and electron microscopy. Proc Natl Acad Sci U S A 103:17903–17908. doi:10.1073/pnas.0608678103. 17093041 PMC1635158

[43] Smith JG, Mothes W, Blacklow SC, Cunningham JM 2004 The mature avian leukosis virus subgroup A envelope glycoprotein is metastable, and refolding induced by the synergistic effects of receptor binding and low pH is coupled to infection. J Virol 78:1403–1410. doi:10.1128/JVI.78.3.1403-1410.2004. 14722295 PMC321377

[44] Matsuyama S, Delos SE, White JM 2004 Sequential roles of receptor binding and low pH in forming prehairpin and hairpin conformations of a retroviral envelope glycoprotein. J Virol 78:8201–8209. doi:10.1128/JVI.78.15.8201-8209.2004. 15254191 PMC446138

[45] Li F, Berardi M, Li WH, Farzan M, Dormitzer PR, Harrison SC 2006 Conformational states of the severe acute respiratory syndrome coronavirus spike protein ectodomain. J Virol 80:6794–6800. doi:10.1128/JVI.02744-05. 16809285 PMC1489032

[46] Roche S, Rey FA, Gaudin Y, Bressanelli S 2007 Structure of the prefusion form of the vesicular stomatitis virus glycoprotein G. Science 315:843–848. doi:10.1126/science.1135710. 17289996

[47] Roche S, Bressanelli S, Rey FA, Gaudin Y 2006 Crystal structure of the low-pH form of the vesicular stomatitis virus glycoprotein G. Science 313:187–191. doi:10.1126/science.1127683. 16840692

[48] Roche S, Gaudin Y 2002 Characterization of the equilibrium between the native and fusion-inactive conformation of rabies virus glycoprotein indicates that the fusion complex is made of several trimers. Virology 297:128–135. doi:10.1006/viro.2002.1429. 12083843

[49] Pertel PE, Fridberg A, Parish ML, Spear PG 2001 Cell fusion induced by herpes simplex virus glycoproteins gB, gD, and gH-gL requires a gD receptor but not necessarily heparan sulfate. Virology 279:313–324. doi:10.1006/viro.2000.0713. 11145912

[50] Fan Q, Lin E, Satoh T, Arase H, Spear PG 2009 Differential effects on cell fusion activity of mutations in herpes simplex virus 1 glycoprotein B (gB) dependent on whether a gD receptor or a gB receptor is overexpressed. J Virol 83:7384–7390. doi:10.1128/JVI.00087-09. 19457990 PMC2708615

[51] Tanaka M, Kagawa H, Yamanashi Y, Sata T, Kawaguchi Y 2003 Construction of an excisable bacterial artificial chromosome containing a full-length infectious clone of herpes simplex virus type 1: viruses reconstituted from the clone exhibit wild-type properties in vitro and in vivo. J Virol 77:1382–1391. doi:10.1128/JVI.77.2.1382-1391.2003. 12502854 PMC140785

[52] Fan Q, Lin E, Spear PG 2009 Insertional mutations in herpes simplex virus type 1 gL identify functional domains for association with gH and for membrane fusion. J Virol 83:11607–11615. doi:10.1128/JVI.01369-09. 19726507 PMC2772692

[53] Dauber B, Pelletier J, Smiley JR 2011 The herpes simplex virus 1 vhs protein enhances translation of viral true late mRNAs and virus production in a cell type-dependent manner. J Virol 85:5363–5373. doi:10.1128/JVI.00115-11. 21430045 PMC3094992

[54] Pomeranz LE, Blaho JA 2000 Assembly of infectious herpes simplex virus type 1 virions in the absence of full-length VP22. J Virol 74:10041–10054. doi:10.1128/JVI.74.21.10041-10054.2000. 11024133 PMC102043

[55] Pasieka TJ, Lu B, Crosby SD, Wylie KM, Morrison LA, Alexander DE, Menachery VD, Leib DA 2008 Herpes simplex virus virion host shutoff attenuates establishment of the antiviral state. J Virol 82:5527–5535. doi:10.1128/JVI.02047-07. 18367525 PMC2395185

[56] Schwartz JA, Lium EK, Silverstein SJ 2001 Herpes simplex virus type 1 entry is inhibited by the cobalt chelate complex CTC-96. J Virol 75:4117–4128. doi:10.1128/JVI.75.9.4117-4128.2001. 11287561 PMC114157

